# How interpersonal power affects empathic accuracy: differential roles of mentalizing *vs*. mirroring?

**DOI:** 10.3389/fnhum.2013.00375

**Published:** 2013-07-19

**Authors:** Dario Bombari, Marianne Schmid Mast, Tobias Brosch, David Sander

**Affiliations:** ^1^Department of Work and Organizational Psychology, University of NeuchâtelNeuchâtel, Switzerland; ^2^Swiss Center for Affective Sciences, University of GenevaGeneva, Switzerland; ^3^Laboratory for the Study of Emotion Elicitation and Expression, Department of Psychology, University of GenevaGeneva, Switzerland

**Keywords:** power, empathic accuracy, mentalizing, mirroring, interpersonal sensitivity

## Abstract

Empathic accuracy (EA)—the correct assessment of the affective states and thoughts of a social partner—affects social behavior and the outcome of interpersonal interactions. Growing evidence has shown that interpersonal power of a perceiver affects EA when assessing a target. This picture, however, is not obvious; there is evidence supporting both the idea that power can improve EA or impair it. Moreover, the mechanisms through which high power individuals are more (or less) accurate at reading others' minds are unknown. The present article provides a new perspective on the power-EA link by investigating how two core abilities involved in EA, mentalizing and mirroring, can explain when and how power is related to EA. The inclusion of findings from neuroimaging studies on mentalizing and mirroring adds a cognitive neuroscience perspective to the power-EA research that has traditionally been conducted in a social psychological framework. The extent to which a given EA-test requires mentalizing or mirroring and the way power affects both of them could explain the contrasting findings. In addition, the analysis of the neural substrates of mentalizing and mirroring may provide new insight into the relationship between power and EA.

## Introduction

Power affects how people perceive their interaction partners (e.g., high power people perceive social interaction partners as a means to an end) (Magee and Smith, 2013) and how they interact with others (e.g., powerful people assert themselves by talking a lot and interrupting others) (Schmid Mast, 2002; Hall et al., 2005). In this contribution, we refer to power interpersonally, as the degree to which an individual can exert control over another person (Schmid Mast et al., 2009). We focus on the psychological properties of power that can be evoked not only by a real hierarchical relationship, but also simply through cues related to power. Other definitions are sometimes used in the literature. Structural power refers to the hierarchical differences in functions or positions (Ellyson and Dovidio, 1985). Status is a group acknowledgment of respect awarded to a specific individual or can be the power derived from membership in a specific social group (Sidanius et al., 2004). Dominance can reflect both an enduring trait of personality (Ellyson and Dovidio, 1985) or a more transient behavior related to the intention of seeking control over others (Schmid Mast, 2002). Because our review focuses on experimentally manipulated power and its effect on EA, the articles we cite define power in a similar way as we do (i.e., control over other people).

Power does not only affect how others are perceived and acted upon; it also affects the degree to which the assessment of a perceiver is correct (Hall et al., under review). Correct assessment of other people's traits and states is called interpersonal sensitivity or interpersonal accuracy (Hall and Bernieri, 2001; Schmid Mast et al., 2012). One aspect of interpersonal sensitivity is empathy, which has been defined as the ability of a perceiver to recognize, understand, and share the emotions, intentions, and feelings of a target (Zaki et al., 2009). Empathic accuracy (EA) is the correspondence between perceiver's judgments and target's states and feelings (Ickes, 1997; Zaki et al., 2009). In the present paper, we investigate the link between power and EA.

Research shows that high power individuals are better at correctly assessing others' emotions and thoughts (Schmid Mast et al., 2009). However, this finding is not unequivocal in that opposite effects have been documented as well (Galinsky et al., 2006). A recent meta-analysis (Hall et al., under review) revealed a small (M *r* = 0.07) but significant effect showing that high power individuals are more interpersonally accurate than low power individuals. It is noteworthy that the way power was operationalized (i.e., dispositional trait, structural power, or experimentally induced power) had no significant effect on this relationship. The huge heterogeneity of the effect sizes extracted from the literature suggests that there are moderators at work, affecting the power-EA link. One such moderator might be the different accuracy tests which require different skills or are sensitive to different underlying cognitive processes. Interpersonal accuracy tests tend to correlate only weakly, if at all, with each other (Hall, 2001; Zebrowitz, 2001), which corroborates the idea that different tests might require different skills or cognitive processing styles.

The mechanism through which power affects accurate interpersonal perception is unknown. Previous studies found that powerful people are more *prosocially oriented* (Cote et al., 2011) but also less *motivated to be accurate* (Stevens and Fiske, 2000) and more *socially distant* (Magee and Smith, 2013). Schmid Mast et al. (2009) showed that *feeling respected and proud* partially explained the high power individuals' greater EA. The meta-analysis by Hall et al. (under review) showed that trait dominance was related to more interpersonal accuracy when measured as empathic/responsible compared to egoistic/aggressive. Another trait aspect of power that might moderate the power-EA link is the implicit need for power (nPower) (Winter, 1973), which can influence the perceived saliency (Schultheiss and Hale, 2007; Wang et al., 2011) and the motivational response (Schultheiss et al., 2008) toward emotional faces. Since people high in nPower are faster at recognizing emotions (Donhauser et al., under review), it is possible that nPower positively affects EA. These examples of the potential mechanisms linking power to EA do not provide a comprehensive explanation of the contrasting findings mentioned above. This is why we propose a framework that might tie together the results of previous studies.

Historically, two approaches have been put forward to explain how we read other people's minds (Goldman and Sripada, 2005). The “theory-theory” explains mindreading as an extraction of meaning from targets' behavior, mental state, and context. The “simulation theory” instead postulates that we understand others through an internal simulation of their mental state. Although these two approaches have been developed quite independently, neuroimaging studies have shown that indeed EA involves two different mechanisms, mentalizing, and mirroring (Zaki et al., 2009) and in the present article, we aim to discuss their potential role in relation to how power affects EA.

*Mentalizing* typically means extracting and understanding another person's goals by making inferences about his/her mind state (Amodio and Frith, 2006; Spunt et al., 2011). It relies on the ability to distinguish between one's own mental perspective and that of others (i.e., theory of mind). Mentalizing skills are often tested through false-belief paradigms where participants read short stories about two characters and need to make inferences about others' minds based on the knowledge available to other people. There is evidence (Lieberman, 2010) that the different mentalizing tasks converge in that they all activate one specific brain area, the dorsomedial prefrontal cortex (dmPFC). Other regions (e.g., the temporo-parietal junction and the temporal pole) might be more contingent on task demands. *Mirroring* typically means simulating the state of the other to understand the content of his/her mind (Zaki and Ochsner, 2012). The rationale is that observing another person activates the corresponding motor and mental representations in the observer, enabling him/her to understand the other's mind (i.e., neural resonance). Mirroring is supposed to rely on the mirror neuron system, which was first discovered in the macaque brain (Gallese et al., 1996). Although the existence of such a system in humans is now quite commonly assumed, the topic is still debated (e.g., Kilner, 2011). According to Lieberman (2010), the mirror system relies on the bilateral posterior ventrolateral PFC and bilateral anterior inferior parietal lobule (IPL). Mirroring is considered a rather automatic, unconscious response based on shared mental representations whereas mentalizing is a rather cognitive aspect of empathy that necessitates an explicit representation of the subjectivity of the social interaction partner (Decety and Jackson, 2004). The two systems cooperate closely, because the mirror system helps provide an early identification of the facial expressions and the mentalizing system processes this input in order to make causal attributions about emotions (Spunt and Lieberman, 2012).

*Accuracy* in assessing others' emotions has been documented to be related to both of the aforementioned brain systems: regions within the mirror neuron system (i.e., the middle frontal gyrus and the IPL) and areas involved in mentalizing (i.e., the superior temporal sulcus and medial PFC) (Zaki et al., 2009). To the extent that the cues about a target's feelings and thoughts become multimodal and dynamic, concurrent activation of both systems might be crucial (Zaki et al., 2009).

In the present article we propose a new perspective on the relationship between power and EA by bringing together two strands of research that have so far been relatively unconnected: the study of power and interpersonal accuracy from a social psychological point of view and the study of EA and its neural bases from the cognitive neuroscience approach. In particular we argue (i) that different EA tasks might require predominantly mirroring or mentalizing skills, (ii) that power might influence both mentalizing and mirroring, and (iii) that power might affect the flexibility to switch between mentalizing and mirroring skills.

## How mirroring and mentalizing may be differently involved in EA tasks: a hypothesis

Different EA tasks may require a perceiver to infer emotions of others, guess what they are thinking, and understand what their intentions and motives are, among others. Mirroring and mentalizing may differently affect each of these aspects. In this section, we illustrate how the tasks used in the studies assessing EA of high and low power people may require mentalizing or mirroring skills (see Table 1 for a summary of the studies on this topic).

**Table 1:** **An overview of the studies investigating the relationship between power and EA**.

**Study**	**Power**		**Setting**	**EA-related assessment**		**Main findings**
	**Manipulation**	**Groups**		**Task**	**Main skills involved**	
Anderson and Berdahl (2002)—Study 1	Role play	High vs. low power	Face-to-face interaction	Difference between two people's ratings	Mirroring + Mentalizing	No significant results
Anderson and Berdahl (2002)—Study 2	Role play	High vs. low power	Face-to-face interaction	Difference between two people's ratings	Mirroring + Mentalizing	Power improves the detection of partners' signals
Boucher et al. (2008)—Study 1	Role play	High vs. low power	Face-to-face interaction	Difference between two people's ratings	Mirroring + Mentalizing	No significant results
Cote et al. (2011)—Study 2	Role play	High vs. low power	Computer-based	Rating of videotaped interactions	Mirroring + Mentalizing	No significant results
Galinsky et al. (2006)—Study 1	Priming (recalling of autobiographical events)	High vs. low power	Computer-based	Perspective taking (draw an E on the forehead)	Mentalizing	Power decreases perspective taking
Galinsky et al. (2006)—Study 2	Priming (recalling of autobiographical events)	High vs. low power	Computer-based	Consideration of communication intentions	Mentalizing	Power decreases perspective taking
Galinsky et al. (2006)—Study 3	Priming (recalling of autobiographical events)	High power vs. control	Computer-based	Emotion recognition (DANVA-2)	Mirroring	Power decreases emotion recognition
Gonzaga et al. (2008)—men	Role play	High, low, and equal power	Face-to-face interaction	Correlation between two people's ratings	Mirroring + Mentalizing	No significant results
Gonzaga et al. (2008)—women	Role play	High, low, and equal power	Face-to-face interaction	Correlation between two people's ratings	Mirroring + Mentalizing	Power decreases EA
Hall et al. (2006)	Role play	High vs. low power	Face-to-face interaction	Decoding non-verbal messages	Mirroring	Power decreases the ability to read partners' signals (due to subordinates' message ambiguity)
Kunstman and Maner (2011)—study 4	Role play	High vs. low power	Face-to-face interaction	Difference between two people's ratings	Mirroring + Mentalizing	Power improves EA
Schmid Mast et al. (2009)—Study 1	Role play	High vs. low power	Computer-based	Rating of videotaped interactions	Mirroring + Mentalizing	Power improves EA
Schmid Mast et al. (2009)—Study 2	Priming (Word completion task)	High and low power, control	Computer-based	Rating of videotaped interactions	Mirroring + Mentalizing	Power improves EA
Schmid Mast et al. (2009)—Study 3	Priming (recalling of autobiographical events)	High and low power, control	Computer-based	Emotion recognition (DANVA-2)	Mirroring	Power improves emotion recognition
Snodgrass (1985)	Role play	High vs. low power	Face-to-face interaction	Correlation between two people's ratings	Mirroring + Mentalizing	Power decreases EA
Snodgrass (1992)	Role play	High vs. low power	Face-to-face interaction	Correlation between two people's ratings	Mirroring + Mentalizing	No significant results

*In the power section, we list how power was manipulated. Role play means the assignment of a participant to a high or low power role in an interaction with a social partner. Priming refers to an implicit manipulation by means of exposure to social cues related to power. In the EA-related assessment section we describe how the components that might influence EA were measured. The setting reports whether EA assessment relied on a face-to-face live interaction or on a computer-based task (e.g., recognition of pictures of emotional expression). In the following column we describe the measure that was used to assess perceivers' behavior. For each study, we report which skills (i.e., mentalizing and/or mirroring) we hypothesize to be predominantly involved in the task that was used. In the last column we report the main finding of the study*.

Some studies used simple recognition of facial expression of emotions to assess EA. This is a very simplistic measure of EA that might not take into account its entire complexity. Simple expression recognition might rely more on mirroring than on mentalizing. Indeed, a number of studies (Dimberg et al., 2000; Hess and Blairy, 2001) found that when participants are presented with pictures of emotional expressions, a facial mimicry response, which is supposed to rely on the mirroring system (Catmur et al., 2008; Heyes, 2011), is automatically elicited. Mentalizing might be less critical than mirroring for facial expression recognition. Even though contrasting findings have been reported (Uljarevic and Hamilton, 2013), some studies showed that children with autism can recognize facial expressions as accurately as typically developing children (Castelli, 2005; Rosset et al., 2008). Autistic children typically have impaired mentalizing skills and the fact that they are able to correctly recognize others' emotions suggests that mentalizing may not play a crucial role in emotion recognition.

In studies in which participants are tested in real-time face-to-face interaction settings, one interaction partner infers the other's feelings during the interaction. Even though this is perhaps a more naturalistic way of testing social variables, there is no control of the mimicry response of participants and therefore of the involvement of the mirroring system. The mimicry response is contingent upon situational factors (e.g., attitudes toward the social target, type of social interaction) and can influence both EA and perceived power. For instance, competitive interactions decrease the mimicry response (Lanzetta and Englis, 1989; Weyers et al., 2009). This could be relevant because hierarchical interactions might be competitive and the inhibition of facial mimicry can in turn impair emotion recognition (Oberman et al., 2007). Moreover, facial mimicry can also influence perceived power: people who mimic more in an interaction are perceived as more likeable (van Baaren et al., 2009) and therefore may be less dominant because likeability (Farley, 2008) and agreeableness (Lippa and Arad, 1999) are negatively related to perceived dominance.

Yet other paradigms might require mentalizing skills to a higher degree. In a paper by Galinsky et al. (2006, Study 1) participants were asked to draw an E on their foreheads right before a live interaction with a partner. Powerful people were less likely to draw the letter by taking the perspective of the interaction partner. Even though perspective taking is not a measure of EA per se, it has been suggested as a mechanism through which power might hamper accuracy in social judgments. Perspective taking can be considered a more inferential type of thinking and might therefore rely mostly on mentalizing skills. Muscatell et al. (2012) found that lower social status was related to greater activity in the mentalizing system while encoding social information. This might explain why low power people engage more in perspective-taking strategies than high power people.

In many of the studies that use experimental manipulation of power, participants are asked to recall autobiographical events related to power (Galinsky et al., 2006; Schmid Mast et al., 2009). With this type of priming, the strategy participants use to recall the events is not controlled, which may represent a confounding factor. Some participants might choose spontaneously to focus on contextual information of the recalled event, a strategy that would foster mentalizing skills and advantage those participants in subsequent tasks requiring inferential reasoning (e.g., perspective taking). This idea is supported by a neuroimaging study by Morelli et al. (2012), which shows that focusing on the context of an emotional event involves the mentalizing system more than the mirroring system. Instead, people focusing more on their own bodily sensations (e.g., the stress of being powerless) might elicit a mirroring response and therefore be more accurate in a subsequent task requiring mirroring (e.g., simple emotion recognition).

Taken together, differences in the tasks used to assess EA and to manipulate power might contribute to explain the contrasting finding concerning their relationship.

## How power may influence mentalizing

Construal Level Theory (CLT; Liberman and Trope, 1998) draws on the concept of psychological distance. Distal entities (e.g., events far in time or space or hypothetical) are more remote from direct experience and therefore need a higher level of construal (i.e., the missing information needs to be taken from more proximal entities). CLT makes specific predictions about power and these have also been taken up by the social distance model of power by Magee and Smith (2013). Powerful people should feel more psychological distance and more dissimilar to powerless individuals (Liberman et al., 2007; Magee and Smith, 2013). Indeed a study by Lammers et al. (2012) provided support for this hypothesis by showing that high power primed people were less willing to collaborate with a social partner on a series of games than low power people. There is evidence that when people are judging targets similar to them, a more ventral region in the medial PFC is activated compared to when people are judging targets that are less similar to them (Mitchell et al., 2006). If high power people perceive low power targets as less similar to them, they might show reduced activation in the ventral medial PFC region, but increased activation in the more dorsal region identified by Mitchell et al. (2006), which correspond to an area typically involved in mentalizing. To the extent that the social distance between high and low power individuals increases, the high power individuals might therefore rely more on mentalizing skills to correctly assess others' thoughts and feelings.

It could also be argued that powerless people rely more on mentalizing than powerful people. Fiske's continuum model of power (Fiske and Neuberg, 1990; Goodwin et al., 2000) predicts that low power people focus their attention on high power people, whereas the latter are more self-focused. A meta-analysis by Denny et al. (2012) showed that a more ventral region of the medial PFC is associated with self-related judgments, whereas a more dorsal region is related to judgments about others. The dmPFC activation suggests mentalizing and indeed its activity is greater in low than high social status people when encoding social information (Muscatell et al., 2012). Further experimental research is therefore necessary in order to specifically test the effects of power on mentalizing.

## How power may influence mirroring

Studies on facial mimicry can support the hypothesis of an influence of power on mirroring. Theories of embodied cognition claim that emotion recognition is achieved through an internal simulation of the perceived expression (Goldman and Sripada, 2005; Niedenthal et al., 2010). Even though mimicry might not be strictly necessary for emotion recognition (Hess and Blairy, 2001; Sander et al., 2007; Rives Bogart and Matsumoto, 2010; Mumenthaler and Sander, 2012), mimicry responses predict the perceived intensity of facial expressions (Sato et al., 2013). Moreover, interfering with mimicry can hamper emotion recognition accuracy (Oberman et al., 2007; Neal and Chartrand, 2011). Research on mimicry is important if we consider that high power individuals tend to be more expressive than low power people in live interactions (Snodgrass et al., 1998). Hsee et al. (1990) found that powerful individuals are more likely to display subordinate's feelings than vice versa. These findings suggest that high power people might engage the mirroring system to a higher degree than low power people when reading others' minds and this might explain their high accuracy on facial expression recognition tests (Schmid Mast et al., 2009, Study 3). In addition, one could argue that low power people mimic less because they might have more negative mood or attitudes (e.g., toward superiors) and this is known to reduce the mimicry response (Likowski et al., 2011). Again, further experimental research is necessary in order to test the effects of power on mirroring.

## Power and flexibility

Hirsh et al. (2011) proposed a model that draws on Keltner et al. (2003) explanations of approach and inhibition. According to these authors, powerful people would be more approach-oriented. This would activate the Behavioral Approach System, which in turn would decrease the conflict between competing responses (i.e., disinhibition). Thus, powerful people would behave according to their most salient response, which can be externally driven, when strong contextual cues are present, or internally driven, in absence of such cues. When the response is internally triggered, power can reveal the internal dispositions of the person. When there are strong contextual cues, Hirsh et al.'s model predicts that powerful people will be more responsive to the affordances required by the task. This is in accordance with the Situated Focus Theory by Guinote (2007), which posits that power fosters the attunement to the situation and increases flexibility. Thus, this suggests that powerful people may be more able to switch flexibly between mentalizing and mirroring skills, according to what is more relevant to task demands.

## Conclusions

Whereas current models of power do not seem to properly account for the heterogeneity found in the literature on the power-EA link, in the present paper we speculate that a focus on the differential role of mirroring vs. mentalizing could. This approach can guide future research in at least two directions. First, empirical studies might test specific hypotheses based on the mechanisms we propose here. We expect that the ambivalent effects of power on EA will be teased apart once the effects of mirroring and mentalizing on power and EA are taken into account. Second, future studies might be more cautious in the choice of the specific EA tasks. Indeed, assessing EA through tasks focusing mostly on mirroring or on mentalizing might dramatically influence the outcomes of a study.

### Conflict of interest statement

The authors declare that the research was conducted in the absence of any commercial or financial relationships that could be construed as a potential conflict of interest.

## References

[B1] Amodio D. M. Frith C. D. (2006). Meeting of minds: the medial frontal cortex and social cognition. Nat. Rev. Neurosci. 7, 268–277 10.1038/nrn1884 16552413

[B1a] Anderson C. Berdahl J. L. (2002). The experience of power: examining the effects of power on approach and inhibition tendencies. J. Pers. Soc. Psychol. 83, 1362–1377 10.1037/0022-3514.83.6.1362 12500818

[B1b] Boucher E. M. Hancock J. T. Dunham P. J. (2008). Interpersonal sensitivity in computer-mediated and face-to-face conversations. Media Psychol. 11, 235–258 10.1080/15213260801906471

[B2] Castelli F. (2005). Understanding emotions from standardized facial expressions in autism and normal development. Autism 9, 428–449 10.1177/1362361305056082 16155058

[B3] Catmur C. Gillmeister H. Bird G. Liepelt R. Brass M. Heyes C. (2008). Through the looking glass: counter-mirror activation following incompatible sensorimotor learning. Eur. J. Neurosci. 28, 1208–1215 10.1111/j.1460-9568.2008.06419.x 18783371

[B4] Cote S. Kraus M. W. Cheng B. H. Oveis C. van der Lowe I. Lian H. (2011). Social power facilitates the effect of prosocial orientation on empathic accuracy. J. Pers. Soc. Psychol. 101, 217–232 10.1037/a0023171 21463075

[B5] Decety J. Jackson P. L. (2004). The functional architecture of human empathy. Behav. Cogn. Neurosci. Rev. 3, 71–100 10.1177/1534582304267187 15537986

[B6] Denny B. T. Kober H. Wager T. D. Ochsner K. N. (2012). A meta-analysis of functional neuroimaging studies of self- and other judgments reveals a spatial gradient for mentalizing in medial prefrontal cortex. J. Cogn. Neurosci. 24, 1742–1752 10.1162/jocn_a_00233 22452556 PMC3806720

[B7] Dimberg U. Thunberg M. Elmehed K. (2000). Unconscious facial reactions to emotional facial expressions. Psychol. Sci. 11, 86–89 10.1111/1467-9280.00221 11228851

[B9] Ellyson S. L. Dovidio J. F. (1985). Power, dominance, and nonverbal behavior: basic concepts and issues, in Power, Dominance, and Nonverbal Behavior, eds Ellyson S. L. Dovidio J. F. (New York, NY: Springer), 1–27 10.1007/978-1-4612-5106-4

[B10] Farley S. (2008). Attaining status at the expense of likeability: pilfering power through conversational interruption. J. Nonverbal Behav. 32, 241–260 10.1007/s10919-008-0054-x

[B11] Fiske S. T. Neuberg S. L. (1990). A continuum of impression formation, from category-based to individuating processes: influences of information and motivation on attention and interpretation, in Advances in Experimental Social Psychology, Vol. 23, ed Zanna M. P. (New York, NY: Academic Press), 1–74

[B12] Galinsky A. D. Magee J. C. Inesi M. E. Gruenfeld D. H. (2006). Power and perspectives not taken. Psychol. Sci. 17, 1068–1074 10.1111/j.1467-9280.2006.01824.x 17201789

[B13] Gallese V. Fadiga L. Fogassi L. Rizzolatti G. (1996). Action recognition in the premotor cortex. Brain 119(Pt 2), 593–609 10.1093/brain/119.2.593 8800951

[B14] Goldman A. I. Sripada C. S. (2005). Simulationist models of face-based emotion recognition. Cognition 94, 193–213 10.1016/j.cognition.2004.01.005 15617671

[B13a] Gonzaga G. C. Keltner D. Ward D. (2008). Power in mixed-sex stranger interactions. Cogn. Emot. 22, 1555–1568 10.1080/02699930801921008

[B15] Goodwin S. A. Gubin A. Fiske S. T. Yzerbyt V. Y. (2000). Power can bias impression processes: stereotyping subodinates by default and by design. Group Process. Intergroup Relat. 3, 227–256 10.1177/1368430200003003001

[B16] Guinote A. (2007). Behaviour variability and the situated focus theory of power. Eur. Rev. Soc. Psychol. 18, 256–295 10.1080/10463280701692813

[B17] Hall J. A. (2001). The PONS Test and the psychometric approach to measuring interpersonal sensitivity, in Interpersonal Sensitivity: Theory and Measurement, eds Hall J. A. Bernieri F. J. (Mahwah, NJ: Lawrence Erlbaum Associates), 143–160

[B18] Hall J. A. Bernieri F. J. (eds.). (2001). Interpersonal Sensitivity: Theory and Measurement. Mahwah, NJ: Lawrence Erlbaum Associates

[B19] Hall J. A. Coats E. J. Smith LeBeau L. (2005). Nonverbal behavior and the vertical dimension of social relations: a meta-analysis. Psychol. Bull. 131, 898–924 10.1037/0033-2909.131.6.898 16351328

[B17a] Hall J. A. Rosip J. C. Smith LeBeau L. Horgan T. G. Carter J. D. (2006). Attributing the sources of accuracy in unequal-power dyadic communication: who is better and why? J. Exp. Soc. Psychol. 42, 18–27 10.1016/j.jesp.2005.01.005

[B21] Hess U. Blairy S. (2001). Facial mimicry and emotional contagion to dynamic emotional facial expressions and their influence on decoding accuracy. Int. J. Psychophysiol. 40, 129–141 10.1016/S0167-8760(00)00161-6 11165351

[B22] Heyes C. (2011). Automatic imitation. Psychol. Bull. 137, 463–483 10.1037/a0022288 21280938

[B23] Hirsh J. B. Galinsky A. D. Zhong C.-B. (2011). Drunk, powerful, and in the dark: how general processes of disinhibition produce both prosocial and antisocial behavior. Perspect. Psychol. Sci. 6, 415–427 10.1177/1745691611416992 26168194

[B24] Hsee C. K. Hatfield E. Carlson J. G. Chemtob C. (1990). The effect of power on susceptibility to emotional contagion. Cogn. Emot. 4, 327–340 10.1080/02699939008408081

[B25] Ickes W. J. (1997). Empathic Accuracy. New York, NY: Guilford Press

[B26] Keltner D. Gruenfeld D. H. Anderson A. (2003). Power, approach, and inhibition. Psychol. Rev. 110, 265–284 10.1037/0033-295X.110.2.265 12747524

[B27] Kilner J. M. (2011). More than one pathway to action understanding. Trends Cogn. Sci. 15, 352–357 10.1016/j.tics.2011.06.005 21775191 PMC3389781

[B27a] Kunstman J. W. Maner J. K. (2011). Sexual overperception: power, mating motives, and biases in social judgment. J. Pers. Soc. Psychol. 100, 282–294 10.1037/a0021135 21142379

[B28] Lammers J. Galinsky A. D. Gordijn E. H. Otten S. (2012). Power increases social distance. Soc. Psychol. Pers. Sci. 3, 282–290 10.1177/1948550611418679

[B29] Lanzetta J. T. Englis B. G. (1989). Expectations of cooperation and competition and their effects on observers' vicarious emotional responses. J. Pers. Soc. Psychol. 56, 543–554 10.1037/0022-3514.56.4.543

[B30] Liberman N. Trope Y. (1998). The role of feasibility and desirability considerations in near and distant future decisions: a test of temporal construal theory. J. Pers. Soc. Psychol. 75, 5–18 10.1037/0022-3514.75.1.5

[B31] Liberman N. Trope Y. Stephan E. (2007). Psychological distance, in Social Psychology: Handbook of Basic Principles, 2nd Edn., eds Kruglanski A. W. Higgins E. T. (New York, NY: Guilford Press), 353–381

[B32] Lieberman M. D. (2010). Social cognitive neuroscience, in Handbook of Social Psychology 5th edn., eds Fiske S. T. Gilbert D. T. Lindzey G. (New York, NY: McGraw-Hill), 143–193

[B33] Likowski K. U. Weyers P. Seibt B. Stöhr C. Pauli P. Mühlberger A. (2011). Sad and lonely? sad mood suppresses facial mimicry. J. Nonverbal Behav. 35, 101–117 10.1007/s10919-011-0107-4

[B34] Lippa R. Arad S. (1999). Gender, personality, and prejudice: the display of authoritarianism and social dominance in interviews with college men and women. J. Res. Pers. 33, 463–493 10.1006/jrpe.1999.2266

[B35] Magee J. C. Smith P. K. (2013). The social distance theory of power. Pers. Soc. Psychol. Rev. 17, 158–186 10.1177/1088868312472732 23348983

[B36] Mitchell J. P. Macrae C. N. Banaji M. R. (2006). Dissociable medial prefrontal contributions to judgments of similar and dissimilar others. Neuron 50, 655–663 10.1016/j.neuron.2006.03.040 16701214

[B37] Morelli S. A. Rameson L. T. Lieberman M. D. (2012). The neural components of empathy: predicting daily prosocial behavior. Soc. Cogn. Affect. Neurosci. 10.1093/scan/nss088 22887480 PMC3871722

[B38] Mumenthaler C. Sander D. (2012). Social appraisal influences recognition of emotions. J. Pers. Soc. Psychol. 102, 1118–1135 10.1037/a0026885 22288528

[B39] Muscatell K. A. Morelli S. A. Falk E. B. Way B. M. Pfeifer J. H. Galinsky A. D. (2012). Social status modulates neural activity in the mentalizing network. Neuroimage 60, 1771–1777 10.1016/j.neuroimage.2012.01.080 22289808 PMC3909703

[B40] Neal D. T. Chartrand T. L. (2011). Embodied emotion perception: amplifying and dampening facial feedback modulates emotion perception accuracy. Soc. Psychol. Pers. Sci. 2, 673–678 10.1177/1948550611406138

[B41] Niedenthal P. M. Mermillod M. Maringer M. Hess U. (2010). The Simulation of Smiles (SIMS) model: embodied simulation and the meaning of facial expression. Behav. Brain Sci. 33, 417–433 discussion: 433–480. 10.1017/S0140525X10000865 21211115

[B42] Oberman L. M. Winkielman P. Ramachandran V. S. (2007). Face to face: blocking facial mimicry can selectively impair recognition of emotional expressions. Soc. Neurosci. 2, 167–178 10.1080/17470910701391943 18633815

[B43] Rives Bogart K. Matsumoto D. (2010). Facial mimicry is not necessary to recognize emotion: facial expression recognition by people with Moebius syndrome. Soc. Neurosci. 5, 241–251 10.1080/17470910903395692 19882440

[B44] Rosset D. B. Rondan C. Da Fonseca D. Santos A. Assouline B. Deruelle C. (2008). Typical emotion processing for cartoon but not for real faces in children with autistic spectrum disorders. J. Autism Dev. Disord. 38, 919–925 10.1007/s10803-007-0465-2 17952583

[B45] Sander D. Grandjean D. Kaiser S. Wehrle T. Scherer K. R. (2007). Interaction effects of perceived gaze direction and dynamic facial expression: evidence for appraisal theories of emotion. Eur. J. Cogn. Psychol. 19, 470–480 10.1080/09541440600757426

[B46] Sato W. Fujimura T. Kochiyama T. Suzuki N. (2013). Relationships among facial mimicry, emotional experience, and emotion recognition. PLoS ONE 8:e57889 10.1371/journal.pone.0057889 23536774 PMC3607589

[B47] Schmid Mast M. (2002). Dominance as expressed and inferred through speaking time: a meta-analysis. Hum. Commun. Res. 28, 420–450

[B48] Schmid Mast M. Jonas K. Cronauer C. K. Darioly A. (2012). On the importance of the superior's interpersonal sensitivity for good leadership. J. Appl. Soc. Psychol. 42, 1043–1068 10.1111/j.1559-1816.2011.00852.x

[B49] Schmid Mast M. Jonas K. Hall J. A. (2009). Give a person power and he or she will show interpersonal sensitivity: the phenomenon and its why and when. J. Pers. Soc. Psychol. 97, 835–850 10.1037/a0016234 19857005

[B50] Schultheiss O. C. Hale J. A. (2007). Implicit motives modulate attentional orienting to perceived facial expressions of emotion. Motiv. Emot. 31, 13–24 10.1007/s11031-006-9042-9

[B51] Schultheiss O. C. Wirth M. M. Waugh C. E. Stanton S. J. Meier E. A. Reuter-Lorenz P. (2008). Exploring the motivational brain: effects of implicit power motivation on brain activation in response to facial expressions of emotion. Soc. Cogn. Affect. Neurosci. 3, 333–343 10.1093/scan/nsn030 19015083 PMC2607053

[B52] Sidanius J. Pratto F. van Laar C. Levin S. (2004). Social dominance theory: its agenda and method. Polit. Psychol. 25, 845–880 10.1111/j.1467-9221.2004.00401.x

[B52a] Snodgrass S. E. (1985). Women's intuition: the effect of subordinate role on interpersonal sensitivity. J. Pers. Soc. Psychol. 49, 146–155 10.1037/0022-3514.49.1.146

[B52b] Snodgrass S. E. (1992). Further effects of role versus gender on interpersonal sensitivity. J. Pers. Soc. Psychol. 62, 154–158 10.1037/0022-3514.62.1.1543766514

[B53] Snodgrass S. E. Hecht M. A. Ploutz-Snyder R. (1998). Interpersonal sensitivity: expressivity or perceptivity? J. Pers. Soc. Psychol. 74, 238–249 10.1037/0022-3514.74.1.238 9457785

[B54] Spunt R. P. Lieberman M. D. (2012). An integrative model of the neural systems supporting the comprehension of observed emotional behavior. Neuroimage 59, 3050–3059 10.1016/j.neuroimage.2011.10.005 22019857

[B55] Spunt R. P. Satpute A. B. Lieberman M. D. (2011). Identifying the what, why, and how of an observed action: an fMRI study of mentalizing and mechanizing during action observation. J. Cogn. Neurosci. 23, 63–74 10.1162/jocn.2010.21446 20146607

[B56] Stevens L. E. Fiske S. T. (2000). Motivated impressions of a powerholder: accuracy under task dependency and misperception under evaluation dependency. Pers. Soc. Psychol. Bull. 26, 907–922 10.1177/01461672002610002

[B57] Uljarevic M. Hamilton A. (2013). Recognition of emotions in autism: a formal meta-analysis. J. Autism Dev. Disord. 43, 1517–1526 10.1007/s10803-012-1695-5 23114566

[B58] van Baaren R. Janssen L. Chartrand T. L. Dijksterhuis A. (2009). Where is the love? The social aspects of mimicry. Philos. Trans. R. Soc. B Biol. Sci. 364, 2381–2389 10.1098/rstb.2009.0057 19620109 PMC2865082

[B59] Wang J. Liu L. Zheng Y. (2011). Effects of implicit power motive on the processing of anger faces: an event-related potential study. J. Res. Pers. 45, 441–447 10.1016/j.jrp.2011.05.004

[B60] Weyers P. Muhlberger A. Kund A. Hess U. Pauli P. (2009). Modulation of facial reactions to avatar emotional faces by nonconscious competition priming. Psychophysiology 46, 328–335 10.1111/j.1469-8986.2008.00771.x 19207205

[B61] Winter D. G. (1973). The Power Motive. New York, NY: Free Press

[B62] Zaki J. Bolger N. Ochsner K. (2009). Unpacking the informational bases of empathic accuracy. Emotion 9, 478–487 10.1037/a0016551 19653768 PMC6558959

[B63] Zaki J. Ochsner K. (2012). The neuroscience of empathy: progress, pitfalls and promise. Nat. Neurosci. 15, 675–680 10.1038/nn.3085 22504346

[B64] Zaki J. Weber J. Bolger N. Ochsner K. (2009). The neural bases of empathic accuracy. Proc. Natl. Acad. Sci. U.S.A. 106, 11382–11387 10.1073/pnas.0902666106 19549849 PMC2708723

[B65] Zebrowitz L. A. (2001). Groping for the elephant of interpersonal sensitivity, in Interpersonal Sensitivity: Theory and Measurement, eds Hall J. A. Bernieri F. J. (Mahwah, NJ: Lawrence Erlbaum Associates), 333–350

